# *Big data* e inteligencia artificial en el futuro manejo de pacientes. ¿Por dónde empezar? ¿En qué punto nos encontramos? ¿Quo tendimus?

**DOI:** 10.1515/almed-2020-0052

**Published:** 2020-08-13

**Authors:** Ashraf Mina

**Affiliations:** NSW Health Pathology, Forensic & Analytical Science Service (FASS), Sydney, Australia; Affiliated Senior Clinical Lecturer, Faculty of Medicine and Health, Sydney University, North Ryde, Australia

**Keywords:** aprendizaje profundo, *big data*, inteligencia artificial, manejo de pacientes, patología digital

## Abstract

**Objetivos:**

Este artículo aborda los aspectos clave e impacto de aplicar el *big data* y la inteligencia artificial para optimizar los modelos de detección temprana, el diagnóstico preciso y rápido, el manejo de pacientes, los tratamientos, la medicina de precisión, y la distribución de recursos.

**Contenido:**

Los procesos de *big data* e inteligencia artificial incluyen el aprendizaje automático, el razonamiento basado en unas reglas para obtener conclusiones aproximadas o definitivas, y la autocorrección. Estos procesos mejorarían la detección de enfermedades, enfermedades raras, toxicidades, e identificarían las causas del infradiagnóstico. El *big data* combinado con la inteligencia artificial (IA), el aprendizaje automático (AA), la computación, la construcción de modelos predictivos y la combinatoria, se emplean para interrogar computacionalmente datos estructurados y no estructurados para detectar patrones, tendencias, y posibles correlaciones y relaciones entre diversas fuentes de datos.

**Resumen:**

Los sistemas de diagnóstico asistido y los dispositivos de salud portátiles no solo se emplearán para el manejo de pacientes, sino también para la prevención y detección temprana de enfermedades. El *big data* también tendrá un impacto para las aseguradoras, fabricantes de dispositivos y compañías farmacéuticas. El *big data* y la Inteligencia Artificial se han diversificado, tienen multitud de aplicaciones y su uso para el seguimiento y diagnóstico se extenderá.

**Perspectivas:**

El *big data*, la conectividad, y la IA de los sistemas de diagnóstico asistido, así como los dispositivos de salud portátiles y los *smartphones* transformarán los métodos tradicionales de manejo de pacientes en la era de la explosión de la información médica.

## Objetivos

Con este artículo se pretende dar a conocer los aspectos clave, importancia, e impacto que la aplicación de la inteligencia artificial y el *big data* en la medicina puede tener en el desarrollo de modelos de detección temprana, el diagnóstico preciso y rápido, el manejo de pacientes, la optimización de los tratamientos, la medicina de precisión y la distribución de recursos. El término *big data* (BD) hace referencia al hecho de que el volumen de datos actualmente disponible es tan ingente, heterogéneo y cambiante que resulta complicado almacenarlos, procesarlos, y extraerles un valor con las tecnologías anteriores [1], [2]. El BD se puede encontrar tanto en formas estructuradas como no estructuradas. Los datos estructurados son datos que encajan claramente en un campo y en columnas fijas en bases de datos y hojas de cálculo relacionales. En las bases de datos relacionales se pueden introducir, buscar, y manipular datos estructurados de forma relativamente rápida. El lenguaje de programación que se emplea para el manejo de datos estructurados recibe el nombre de lenguaje de consulta estructurado (*SQL* por sus siglas en inglés). Este lenguaje fue desarrollado por IBM a principios de los años setenta, y resulta de especial utilidad a la hora de manejar relaciones en bases de datos [3].

Aunque los datos estructurales nos ofrecen una perspectiva de los procesos en curso y de resultados clínicos, los datos no estructurados nos pueden ayudar a comprender con mayor profundidad comportamientos, asociaciones, tendencias y propósitos. La revolución No-solo-SQL (*NoSQL*, por sus siglas en inglés) sugiere que podemos hallar un significado oculto en datos no estructurados, sin necesidad de tener que darles una estructura creando modelos para obtener datos relacionales.

Los datos no estructurados no se pueden procesar y analizar con herramientas convencionales. Algunos ejemplos de datos no estructurados son los archivos de texto, los datos de vídeos médicos obtenidos con dispositivos médicos de obtención de imágenes (e. g., endoscopio, laparoscopio, robot quirúrgico, cápsula endoscópica, videocámaras de emergencia, toracoscopio, etc.), datos de bioseñales que se han mostrado en la pantalla de monitorización de los pacientes en los quirófanos o en las unidades de cuidados intensivos y en los dispositivos portátiles de vigilancia de la salud, datos de audio creados verbal y no verbalmente a partir de los pacientes y el personal médico. También se incluye la actividad en el teléfono móvil y en las redes sociales, la actividad de los sensores, los datos de geolocalización, las imágenes satélite y de vigilancia, así como las páginas web. Un impresionante 80% de la totalidad de los datos generados hoy en día se consideran no estructurados y este número seguirá aumentado conforme el número de dispositivos conectados a la Red siga creciendo [4].

## Introducción

El BD y la IA brindan un enorme abanico de posibilidades, aunque también plantean enormes dificultades. En un informe titulado *Cruzando el abismo de la calidad*, el Institute of Medicine de los EE.UU ha identificado seis objetivos para mejorar la calidad de la atención sanitaria. Estos objetivos son lograr una medicina segura, efectiva, centrada en el paciente, eficiente, y equitativa. Estas seis dimensiones se pueden mejorar aprovechando los recursos que el BD ofrece. Así mismo, el BD se puede emplear para evaluar retrospectivamente la calidad de la asistencia, y ayudar a los médicos a ofrecer una atención de alta calidad en tiempo real [5]. Para integrar esta tecnología, con un flujo constante y continuo de datos a capturar y analizar en tiempo real, es preciso contar con una plataforma tecnológica multidisciplinar, lo que supone un cambio sustancial con respecto a la tradicional estructura de los diferentes niveles del sistema sanitario, incluyendo la gestión, la informática, la captación de talentos, los flujos de trabajo, la interpretación de los resultados clínicos y su impacto en la toma de decisiones.

El BD, la IA, y el aprendizaje automático (AA) se podrán aplicar en diferentes áreas sanitarias, transformando el manejo de los pacientes. Cuando se combina el BD y la IA/AA, se pueden hallar respuestas que permitirán reducir costes, ahorrar tiempo, desarrollar nuevos productos y optimizar ofertas de productos, una toma de decisiones inteligente, una planificación comercial estratégica, determinar la causa última de los fracasos, problemas o defectos casi en tiempo real, recalcular carteras de riesgo en cuestión de minutos, y detectar conductas ilegítimas o fraudulentas [6].

## Grandes inversiones en salud de organizaciones no médicas

Las ciencias de la vida se beneficiarán de los avances logrados en el manejo de BD por gigantes tecnológicos como Google, Amazon, Apple, Microsoft y Facebook, que cuentan con el poder de la información.

Google aspira a liderar la innovación en salud aprovechando sus potentes capacidades en IA y AA, para interpretar vastos conjuntos de datos médicos. Estas dos tecnologías, que son la infraestructura básica necesaria para el manejo de datos, son aplicables al sector sanitario. Según datos del Centre for Medicare and Medicaid Services (CMS), se espera que los 3000 millones de dólares que los EE.UU tuvieron de gasto sanitario en 2016 vayan aumentando de media un 5,5% anual hasta 2026 [7]. De hecho, con más de 7000 millones de dólares anuales, el gasto sanitario ya representa casi un 10% del PIB [8].

Los dispositivos y aplicaciones de móvil de Apple permiten a los pacientes informarse mejor sobre sus enfermedades o tratamientos, del mismo modo que los médicos pueden consultar los resultados de las analíticas y las imágenes radiológicas, y las enfermeras pueden utilizar aplicaciones para enviar y recibir mensajes de forma segura, ayudando a garantizar la seguridad del paciente a la hora de administrarles la medicación [9]. Apple Watch es capaz de detectar un latido irregular (esto es, detectar una fibrilación auricular), realizar un seguimiento del Parkinson, realizar pruebas de visión, auditivas, o detectar problemas en el habla asociados al ictus, y controlar la diabetes. Así mismo, la interfaz de programación de aplicaciones HealthKit de Apple está ayudando a la compañía desarrolladora de la aplicación Medisafe, que personaliza la tecnología para ayudar a los pacientes a gestionar su medicación, a abordar el problema de la falta de adherencia a tratamiento, que representa un coste de 300.000 millones de dólares.

La división IBM Watson Health fue creada para ayudar a los profesionales e investigadores sanitarios de todo el mundo a darle un sentido a los datos y al conocimiento, con el fin de poder tomar decisiones más informadas en el manejo de los pacientes. Watson Health está dando soporte a las unidades de oncología de más de 300 hospitales y organizaciones sanitarias, existiendo una evidencia cada vez mayor de su utilidad en el campo de la medicina [10].

Amazon ha creado un equipo llamado Grand Challenge, que está trabajando en una serie de audaces proyectos de investigación oncológica, historiales médicos, y la distribución de última milla. Amazon, JP Morgan Chase Company, y Berkshire Hathaway Company han anunciado la formación de una nueva compañía médica que equipará a sus empleados con soluciones tecnológicas para que puedan proporcionar una atención de calidad a un precio razonable, sin incentivos de lucro. Existen alrededor de 1,2 millones de empleados, combinados, repartidos en diferentes mercados, lo cual se traduce en un vasto volumen de datos.

En 2017, Microsoft creó un departamento de salud en su laboratorio de investigación de Cambridge para investigar las aplicaciones de la IA, el AA, y la computación en la nube para introducirse en el sector sanitario. Las soluciones para la monitorización de pacientes, así como la investigación sobre la diabetes son algunas de las áreas de interés del gigante tecnológico en su búsqueda por mejorar la atención sanitaria.

En 2017, Facebook registró la patente de un algoritmo que trata de analizar las emociones de los usuarios por la manera en que escriben en comparación con su forma habitual de teclear. Si la gente aporrea el teclado de su teléfono con más fuerza o teclea con más lentitud de lo habitual, esto podría indicar que están enfadados o deprimidos [11].

## Datos del mundo real y evidencia del mundo real

Los datos del mundo real (DMR) son datos procedentes de diferentes fuentes, asociados a resultados clínicos en una población heterogénea de pacientes en entornos del mundo real [12]. Esto significa que se podrán establecer relaciones y asociaciones, en tanto en cuanto se pueda vincular una serie de datos sobre el paciente, como sus síntomas, patología, pruebas radiológicas, notas clínicas, historias clínicas electrónicas, facturas a aseguradoras, bases de datos de facturación, registros, datos generados por el paciente y su dispositivo móvil, entre otra información relevante, con un resultado clínico, como un diagnóstico, fallecimiento, recurrencia o cualquier otro resultado. La integración de datos genómicos con otras fuentes de datos del mundo real (DMR) podría permitir una mayor estratificación de las poblaciones, aprovechando múltiples indicadores clínicos y algoritmos de construcción de modelos [13]. Se puede hacer un estudio combinado de diagnósticos clínicos, resultados de analíticas, e información genómica para identificar y estratificar subpoblaciones de pacientes y ayudar a identificar biomarcadores, realizar análisis predictivos, o realizar estudios prospectivos.

En el campo de la medicina, la evidencia del mundo real (EMR) es la evidencia obtenida a partir de DMR, que son datos observacionales obtenidos fuera del contexto de los ensayos clínicos controlados aleatorizados, y generados durante la práctica clínica habitual. Para evaluar los resultados clínicos de los pacientes y garantizar que a los pacientes se les administra el mejor tratamiento para ellos, hace falta utilizar DMR. La EMR se genera analizando datos almacenados. Se puede obtener a partir de estudios observacionales retrospectivos o prospectivos y de registros observacionales. En EE.UU, la ley 21 Century Cures Act establece que la FDA debe dar más relevancia a la EMR.

La EMR entra en juego cuando en un ensayo clínico no se puede representar a la totalidad de la población de pacientes de una patología concreta. Los pacientes con comorbilidades, o que pertenecen a una región geográfica remota, o que se encuentran lejos del límite de edad, que no participaron en ningún ensayo clínico pueden no responder al tratamiento en cuestión como se esperaba.

## *Big data* y la industria farmacéutica

Las entidades reguladoras consideran los ensayos clínicos aleatorizados (ECAs) la piedra angular a la hora de evaluar la seguridad y efectividad de los fármacos, aunque su coste, duración y limitado potencial de extrapolación han forzado a algunos a dirigir la mirada hacia la EMR, basada en datos que no se han obtenido en un ECA.

Los registros y bases de datos sanitarias longitudinales pueden reemplazar en algunos casos a los ECAs. Cabe mencionar que algunos estudios basados en este tipo de bases de datos no han funcionado. Para entender la razón por la que el uso de *big data* no ha funcionado para la toma de decisiones regulatorias habría que responder a dos cuestiones relativas a la investigación. La primera es cuándo podemos estudiar los efectos de un fármaco sin aleatorización (controlada por factores externos, no por los investigadores), y la otra es cómo hacer que un análisis de EMR válido pueda reemplazar a un ECA y cómo evitar errores en dicho análisis [14].

BD puede acelerar y acelerará el proceso de desarrollo de fármacos mediante la identificación e interrogación de cohortes en tiempo real, analizando comorbilidades y otros datos demográficos sin la necesidad de tener que realizar un costoso proceso de reclutamiento de pacientes, obtener consentimientos, y secuenciar muestras. La EMR consiste en obtener datos en entornos diferentes a los ensayos clínicos aleatorizados tradicionales, existiendo un creciente interés en este campo.

## *Big data* y las enfermedades raras

Las enfermedades raras ya no son tan raras, ya que 350 millones de personas en todo el mundo padecen una enfermedad rara, se han identificado 700 enfermedades raras, el 50% de los pacientes con enfermedades raras son niños, el 40% de los pacientes reciben un diagnóstico inicial erróneo, se consulta a una media de 7,3 médicos antes de recibir un diagnóstico, y se tarda una media de 4,8 años en obtener un diagnóstico preciso [15].

Rara vez reciben estos pacientes un diagnóstico temprano, ya que puede resultar complicado. En consecuencia, la enfermedad puede empeorar antes de haber obtenido un diagnóstico, y los pacientes podrían haber recibido un tratamiento efectivo que podría haber funcionado en un estadio de la enfermedad menos avanzado. El BD y la IA es capaz de predecir patrones complejos y sutiles a la hora de diagnosticar a los pacientes, e incluso predecir nuevos pacientes cuya enfermedad no había sido identificada. Aplicar DMR sobre sintomatología, diagnósticos, historial de tratamiento, pruebas diagnósticas, etc. combinando algoritmos de reconocimiento de patrones, para facilitar el desarrollo de técnicas de reconocimiento de patrones ayudará a obtener el diagnóstico rápido de una enfermedad rara e identificar a otros pacientes a los que aún no se haya diagnosticado.

IMS Health (IMS Health y Quintiles, actualmente denominados IQVIA) cuenta con una base de datos de DMR de más de 800 millones de pacientes anónimos. IQVIA combinó DMR con la analítica predictiva para ayudar a detectar casos no diagnosticados de enfermedades raras. Un equipo de bioestadísticos, científicos de datos, y expertos clínicos aplicaron modernos métodos de AA entre los que se encontraban análisis predictivos para analizar datos de 70.000 pacientes seleccionados aleatoriamente aplicando un algoritmo inicial basado en la puntuación de riesgo, obteniendo un grupo de alto riesgo que incluía el 8% de los casos confirmados. A continuación, mediante otro algoritmo de AA, se calculó una puntuación de riesgo que arrojó una prevalencia del diagnóstico confirmado del 20,5%. Sin embargo, solo el 0,7% de los pacientes del grupo de muestra padecía la enfermedad. Este estudio aportó evidencia de que el uso de un algoritmo puede aumentar las probabilidades de identificar antes a pacientes de alto riesgo [16].

## *Big data* que identifica las barreras del sistema causantes de un infradiagnóstico

En otro estudio, IQVIA desarrolló un algoritmo de selección de cohortes que aplicaba datos estadísticos de episodios hospitalarios, que incluían datos sobre pacientes ambulatorios, hospitalizados, así como de urgencias, atendidos en Inglaterra en un periodo de cinco años. El estudio reveló que, en los tres años anteriores al diagnóstico formal, los pacientes habían presentado un elevado número de eventos, con el 90% de los pacientes habiendo sido atendidos en el hospital en dicho periodo. Además, el estudio desveló una variabilidad considerable en el índice de incidencias por cada 100.000 habitantes, lo que sugiere un infradiagnóstico en algunos centros terciarios [16].

## El papel de los dispositivos portátiles y *smartphones* en el manejo de pacientes

Cada vez existen más dispositivos y sensores portátiles en el mercado, que representan una buena oportunidad de negocio para los fabricantes. Los dispositivos portátiles, susceptibles de ser adquiridos por un gran número de usuarios, son un negocio mucho más rentable que vender unos pocos equipos médicos.

Cuando se lanzaron al mercado los primeros dispositivos portátiles, estos aún tenían un uso recreativo, habiendo evolucionado rápidamente a un uso científico y, finalmente, clínico [17], [18], [19], [20], [21], [22]. A continuación ofrecemos algunos ejemplos para ilustrar mejor estas aplicaciones. Los dispositivos que se pueden llevar en la muñeca, como el Apple Watch Series 2, Samsung Galaxy Gear S3, y Fitbit Charge 2, miden con precisión la frecuencia cardíaca de taquiarritmia supraventricular basal e inducida [19]. Del mismo modo, los usuarios pueden llevar unos monitores de glucosa aprobados por la FDA en los EE.UU, conectados a aplicaciones digitales, conectadas a su vez directamente a su servicio de salud. En los próximos años, existirá un mayor volumen de datos de salud obtenidos fuera del sistema sanitario que dentro. Adamant Technologies ha creado un chip para ordenadores que detecta el olor y el sabor y los digitaliza, lo cual significa que los *smartphones*, ordenadores o dispositivos podrán oler por sí mismos. Algunas aplicaciones de esta tecnología son el seguimiento metabólico, el seguimiento de patologías como el asma, la diabetes, la medición de niveles de alcohol en sangre, e incluso la detección del cáncer. El Advanced Institute of Science and Technology (KAIST) de Corea ha desarrollado un método rápido y efectivo para diagnosticar enfermedades como la diabetes o el cáncer de pulmón. Este dispositivo utiliza un sensor de exhalación de alta sensibilidad que se puede montar en el *smartphone*. Hecho de nanofibras de dióxido de estaño recubiertas de nanopartículas catalíticas de platino, el sensor es capaz de detectar la presencia de acetona (una señal de diabetes) o tolueno (una señal de cáncer de pulmón), incluso en concentraciones inferiores a las 100 partes por cada 1.000 millones. Otro ejemplo es el sujetador inteligente iTBra desarrollado por Cycardia Health, que realiza mensualmente un escaneo de la mama. Cada tres minutos, una mujer es diagnosticada de cáncer de mama.

## Conclusiones

Aunque el concepto y las aplicaciones de BD en el sector sanitario aún están en fase inicial, este ayudará a comprender mejor un buen número de enfermedades y permitirá explotar datos que hasta ahora no se venían utilizando, permitiendo así mejorar la calidad de la atención sanitaria. Además, las tecnologías de BD podrán explotar la creciente disponibilidad de datos electrónicos de historiales clínicos electrónicos, ensayos clínicos, aplicaciones electrónicas de salud, así como datos genómicos, transcriptómicos, proteómicos, metabólicos y microbiómicos, permitiendo mejorar la rentabilidad de los servicios sanitarios, los modelos predictivos de curso de la enfermedad y respuesta a terapia, la caracterización de heterogeneidad de la enfermedad, la seguridad de los fármacos, y desarrollar así una medicina de precisión [23], [24], [25], [26]. El BD tiene el potencial de mejorar la atención sanitaria y reducir costes a través de la medicina personalizada, combinando múltiples fuentes de datos sobre un paciente concreto.

Cuanto más se utilice el BD en las ciencias médicas y conforme se vayan construyendo sofisticados algoritmos para el análisis de causalidad, más específicos serán los modelos, de acuerdo con las necesidades de cada aplicación [27]. A modo ilustrativo, se ha utilizado el BD en las transfusiones para detectar complicaciones asociadas a las mismas, determinando patrones de uso de sangre, identificando tendencias en los planes de solicitud de sangre para cirugía, y en la evaluación comparativa. Además, el BD puede servir para supervisar la adherencia mediante una serie de indicadores de rendimiento, para el inventariado y gestión general de sangre, y optimizar así el empleo de la misma [28].

Research from International Data Corporation (IDC), una agencia internacional de investigación de mercado, afirma que las compañías que cuenten con los datos adecuados incrementarán su productividad aumentando sus ganancias en 430 mil millones de dólares en 2020. Así, no sorprende que IBM calcule que en los próximos años se van a ofrecer unos 2,72 millones de puestos de trabajo relacionados con la ciencia de datos [29]. Sobre un tercio de los datos mundiales lo genera la industria sanitaria. Las características del BD se resumen en las seis uves: velocidad, variedad, veracidad y variabilidad, que son importantes factores a tener en cuenta. La analítica de BD en medicina y salud consiste en la integración y análisis de grandes volúmenes de complejos datos heterogéneos, tales como datos de las ciencias ómicas (genómica, epigenómica, transcriptómica, proteómica, metabolómica, interactómica, farmacogenómica, diseasómica), datos biomédicos y datos de los historiales médicos electrónicos [24].

Las áreas de IA, AA, y aprendizaje profundo han sido objeto de un creciente interés en los últimos dos años. AA es un subtipo de IA consistente en la creación de algoritmos que se modifican automáticamente sin intervención humana, para producir una información a partir de datos estructurados. El aprendizaje profundo (AP) es un subtipo de AA en el que se crean algoritmos cuyo funcionamiento es similar al del AA, aunque estos algoritmos contienen numerosas capas, cada una de las cuales aporta una interpretación diferente de los datos introducidos. Esta red de algoritmos recibe el nombre de redes neuronales artificiales, ya que su funcionamiento imita el de las redes neuronales humanas del cerebro [30].

Las redes neuronales convolucionales profundas han supuesto una revolución en el procesamiento de imágenes, videos, locuciones y grabaciones, mientras que las redes neuronales recurrentes han arrojado luz sobre datos secuenciales, como los de un texto o un discurso. El AP permite desarrollar modelos computacionales compuestos de múltiples capas de procesamiento que aprenden a representar datos con múltiples niveles de abstracción. Estos métodos han permitido mejorar sustancialmente las tecnologías de reconocimiento de voz y de objetos visuales, la detección de objetos, así como muchas otras áreas como el descubrimiento de fármacos y la genómica. El AP descubre estructuras intrincadas en enormes conjuntos de datos, empleando el algoritmo de retropropagación para indicar los cambios que debe hacer una máquina en los parámetros internos que utiliza para computar la representación en cada capa, a partir de la representación en la capa anterior. El AP requiere de un volumen mucho mayor de datos que un algoritmo de AA tradicional. La razón es que el AP solo es capaz de identificar conceptos y diferencias en capas de redes neuronales cuando se expone a más de un millón de entradas de datos. Los algoritmos de AA, por otro lado, son capaces de aprender a través de una serie de criterios preconfigurados [2].

La aplicación de AA en la patología computacional, que se define como una estrategia diagnóstica basada en el uso de múltiples fuentes de datos (e. g., patología, radiología, clínica, molecular y analítica) aporta conocimiento práctico para médicos, investigadores y pacientes [31]. La cultura de trabajo también jugará un papel importante, al ser necesario un enfoque más inclusivo, que requiere de la cooperación de diferentes expertos de diversas áreas, entre los que se encuentran los especialistas en aplicaciones informáticas, expertos médicos y científicos, científicos de datos, ingenieros de sistemas y hardware, bioestadísticos, y diseñadores de algoritmos.

Hacia 2020, se calcula que la información disponible sobre el organismo, la salud y la medicina se duplique cada 73 días. De hecho, para mantenerse al tanto de toda la literatura científica sobre atención primaria, un médico de familia tendría que leer durante unas 21 horas al día. Algunos autores sugieren que la única manera de que los profesionales médicos puedan mantenerse al día sobre la creciente información disponible, habría que integrar tecnologías como la de los sistemas de soporte a la toma de decisiones clínicas [32]. Sin embargo, se han planteado algunas cuestiones, como que no todos los médicos están a favor de los cambios y no se sienten cómodos con que se tomen decisiones médicas empleando sofisticados modelos basados en el BD y la IA, en lugar de apoyarse exclusivamente en la experiencia del facultativo.

Entre 2011 y 2016, los errores diagnósticos y las complicaciones quirúrgicas en los hospitales públicos supusieron al estado de Nueva Gales del Sur en Australia un coste de más de 262 millones de dólares [33]. Los costes se pueden minimizar mediante mejores medidas preventivas, con terapias mejor dirigidas, y mejorando la adherencia al tratamiento. La intervención temprana en el curso de la salud de un paciente antes de que pase a un estado patológico ahorraría costes derivados de hospitalizaciones imprevistas, consultas en urgencias, y citas en el médico.

## Perspectivas

El BD también tiene implicaciones evolutivas y revolucionarias en el campo de la epidemiología, permitiendo la identificación e intervención sobre factores determinantes de la salud de la población [34]. La revolución del BD mejorará sustancialmente la granularidad y los plazos de publicación de información epidemiológica, con sistemas híbridos que mejorarán, en lugar de sustituir, los tradicionales sistemas de vigilancia, con mejores perspectivas para los modelos y previsiones de enfermedades infecciosas. En psiquiatría, el análisis de BD brindará una oportunidad sin precedentes de exploración, observación descriptiva, generación de hipótesis, y predicción. Los resultados de los estudios de BD serán trasladados a la práctica clínica. Aún quedan algunas dificultades técnicas relacionadas con la calidad, el análisis, y la gestión de BD [35]. A nivel internacional, puede que sea preciso crear una organización internacional que establezca los criterios y la base para el intercambio, almacenamiento, ética, privacidad, y seguridad de los datos. Con vistas al futuro, las facultades médicas y de diversas ciencias deberían ampliar sus programas de estudios en lugar de limitarse a enseñar estadística, e integrar la IA, AA, AP, la computación, la creación de modelos predictivos, y la combinatoria.

Las aplicaciones y los dispositivos portátiles de salud ya son conocidos y ampliamente utilizados por la población, y pueden ser de apoyo con el propósito de lograr una mayor implicación en la gestión de la salud. Para superar barreras y crear conciencia sobre la privacidad y confidencialidad de los datos, es necesaria la implicación de pacientes y médicos [18], [36]. La oleada de datos sobre enfermedades y fármacos ha facilitado la aplicación de metodologías computacionales para transformar el descubrimiento de fármacos. Se podrían aprovechar diversos recursos y herramientas para llevar a cabo dichos análisis [37].

La privacidad es un aspecto que suscita gran preocupación en el intercambio de datos, aunque con la tecnología de seguridad adecuada, y enmascarando los datos personales de los pacientes, se podría superar esta limitación [38]. Aunque hablamos de un volumen de datos mucho mayor, el mismo problema de seguridad se plantea actualmente con respecto a los datos. El Reglamento General de Protección de Datos (RGPD) es una normativa europea sobre la protección y privacidad de las personas en lo que respecta a sus datos personales. Conforme se vaya generalizando el uso de BD, será importante mantener la confianza de la población salvaguardando la seguridad, gobernanza, y confidencialidad de los datos [39].

Los seres humanos vivimos ahora más que nunca y queremos participar en las decisiones sobre cómo y dónde vamos a ser atendidos. La población no solo espera obtener buenos resultados clínicos, sino tener una mejor experiencia, acceder a una medicina basada en la prevención, no únicamente en el tratamiento, y tener una atención más personalizada cerca de casa. Los avances en el análisis de datos ayudarán a influir en la actitud de la comunidad en temas prioritarios como la obesidad infantil, la diabetes, la resistencia a los antibióticos, el diagnóstico de enfermedades raras y los modelos predictivos, e incluso ayudarán a detectar y resolver crímenes. Las tecnologías descritas en el presente artículo no solo suponen un salto en la tecnología aplicada a la salud, sino que son también una tecnología revolucionaria que cambiará la forma en que se diseñan y prestan los servicios de atención sanitaria.
